# Ambient Stable Quantitative PCR Reagents for the Detection of *Yersinia pestis*

**DOI:** 10.1371/journal.pntd.0000629

**Published:** 2010-03-09

**Authors:** Shi Qu, Qinghai Shi, Lei Zhou, Zhaobiao Guo, Dongsheng Zhou, Junhui Zhai, Ruifu Yang

**Affiliations:** Laboratory of Analytical Microbiology, State Key Laboratory of Pathogen and Biosecurity, Beijing Institute of Microbiology and Epidemiology, Beijing, China; University of Maryland School of Medicine, United States of America

## Abstract

**Background:**

Although assays for detecting *Yersinia pestis* using TaqMan probe-based real-time PCR have been developed for years, little is reported on room-temperature-stable PCR reagents, which will be invaluable for field epidemic surveillance, immediate response to public health emergencies, counter-bioterrorism investigation, etc. In this work, a set of real-time PCR reagents for rapid detection of *Y. pestis* was developed with extraordinary stability at 37°C.

**Methods/Principal Findings:**

TaqMan-based real-time PCR assays were developed using the primers and probes targeting the 3a sequence in the chromosome and the F1 antigen gene *caf1* in the plasmid pMT1of *Y. pestis*, respectively. Then, carbohydrate mixtures were added to the PCR reagents, which were later vacuum-dried for stability evaluation. The vacuum-dried reagents were stable at 37°C for at least 49 days for a lower concentration of template DNA (10 copies/µl), and up to 79 days for higher concentrations (≥10^2^ copies/µl). The reagents were used subsequently to detect soil samples spiked with *Y. pestis* vaccine strain EV76, and 5×10^4^ CFU per gram of soil could be detected by both 3a- and *caf1*-based PCR reagents. In addition, a simple and efficient method for soil sample processing is presented here.

**Conclusions/Significance:**

The vacuum-dried reagents for real-time PCR maintain accuracy and reproducibility for at least 49 days at 37°C, indicating that they can be easily transported at room temperature for field application if the machine for performing real-time PCR is available. This dry reagent is of great significance for routine plague surveillance.

## Author Summary

Plague, caused by *Yersinia pestis*, is one of the oldest and most dangerous diseases in human history, and has claimed millions of lives in the three major historical pandemics. Although panic caused by the Black Death is fading, the threat of the reemergence of plague pandemics still exists, with the additional potential of misuse in biowarfare or bioterrorism. Rapid on-site detection and identification of the pathogen is of paramount significance for timely implementation of effective countermeasures. TaqMan probe-based real-time PCR assays can give quick and accurate identification; however, the need for cold delivery and storage prevents its potential on-site application. The objective of this study was to develop a stable PCR system for easy delivery and storage under room temperature, which is vital for conventional plague surveillance and for preparedness in public health emergencies. We present a solution to this particular issue, hoping that it is helpful to future applications.

## Introduction

*Yersinia pestis*, the causative pathogen of the plague, mainly resides in rodents and can be transmitted to humans by infected fleas [1]. As *Y. pestis* is highly virulent and infectious, it has always been recognized as one of the classical biological warfare agents [2] and was classified as a Category A pathogen by the U. S. Center for Disease Control and Prevention (http://www.bt.cdc.gov/agent/agentlist-category.asp) [3].

*Y. pestis* was traditionally identified by bacterial isolation and microscopy observation [4], the phage lysis assay [5] and animal experiments, which was termed as a “four-step” protocol in China. Although it is time-consuming and laborious, this protocol is still a gold standard for laboratory confirmation of *Y. pestis* infections. Immunological methods were also developed for the detection of F1 antigen and antibodies against *Y. pestis* [6],[7],[8]. Immunological biosensors, based on fiber optics, magnetic and up-converting phosphor technology, were recently applied in detection of antigen and antibodies of *Y. pestis* as well [9],[10],[11]. These methods have played important roles in fighting plague, however, nucleic acid-based detection techniques could be an even powerful alternative for detecting *Y. pestis*.

Conventional PCR-gel electrophoresis method has been developed for detecting *Y. pestis* in fleas and other specimens [12],[13],[14]. A handful of real-time quantitative PCR assays in various formats were also established for detecting and identifying *Y. pestis* [15],[16],[17],[18],[19],[20],[21],[22] [23]. Real-time PCR assays provide greater specificity, and they require less time and labor to complete than conventional PCRs. The techniques applied include SYBR Green [24], molecular beacon [25], TaqMan probes [18],[20] and minor groove binding (MGB) probes [22] ect., targeting specific sequences on the chromosome and (or) plasmids. Although real-time PCR has been successfully used in detecting and identifying *Y. pestis*, the relevant reagents need to be transported under low temperature in dry ice in order to keep the activities of enzymes and labeled probes. In this report we developed a room-temperature stable reagent for real-time PCRs which targeted the 3a sequence [26] in chromosome and the *caf1* gene [14] in the plasmid pMT1. This reagent could be stable during transportation at room temperature and thus be reliably applied for on site detection of target microorganisms if the thermal cycler is available.

## Materials and Methods

### Genomic DNAs

Genomic DNAs of four biovars (*Microtus*, *Orientalis*, *Antiqua* and *Mediaevalis*) of *Y. pestis* were stored in our lab. Closely related or other genomic DNAs used in this study include 9 species of *Yersinia* (*Y. enterocolitica*, *Y. intermedia*, *Y. aldovae*, *Y. bercovieri*, *Y. frederiksenii*, *Y. kristensenii*, *Y. mollaretii*, *Y. rohdei*, *Y. ruckeri*), 16 different serotypes of *Y. pseudotuberculosis*, *Brucella*, *Francisella tularensis*, *Bacillus anthracis*, and *E. coli* DH5α; and DNAs from human blood and mouse. All bacterial strains used in this study were listed in Table 1.

**Table 1:** Strains used in this study.

Species	Strain*	Biovar or Serotype
*Yersinia pestis*	49006	*Antiqua*
	47004	*Mediaevalis*
	82009	*Orientalis*
	18014	*Microtus*
*Yersinia pseudotuberculosis*	ATCC 29833	O:1a
	PTB1	O:1b
	Kuratani	O:1c
	53519	O:2
	1134	O:3
	CBSLAM1684	O:4a
	53522	O:5
	DD110	O:6
	141	O:7
	151	O:8
	R708Ly	O:9
	6088	O:10
	R80	O:11
	MW864-2	O:12
	CBSLAM1695	O:13
	93422	O:15
*Y.aldovae*	ATCC 35236	
*Y.bercovieri*	ATCC 43970	
*Y.enterocolitica*	ATCC 9610	
*Y.frederiksenii*	ATCC 33641	
*Y.intermedia*	ATCC 29909	
*Y.kristensenii*	ATCC 33638	
*Y.mollaretii*	ATCC 43969	
*Y.rohdei*	ATCC 43380	
*Y.ruckeri*	ATCC 29473	
*Brucella.melitensis*	28411	
*Francisella tularensis*	410101	
*Bacillus anthracis*	*Sterne*	
*E. coli*	*DH5α*	

*All strains were collected in our lab.

### Primers and probes

The primers and probes for real-time PCR were designed based on the 3a sequence in the chromosome [26] and *caf1* in plasmid pMT1 using Primer Express 2.0 (PE Corporation,USA). Other primers were also designed for cloning the target 3a and *calf1* sequences into pGEM-T Easy Vector (Promega, USA). The primers and probes used in this study were listed in Table 2.

**Table 2:** List of primers and probes used in this study.

Primers and probes	Sequence( 5′- 3′)^d^	Location^e^	Length of the Amplicon(bp)	Comments
3a-CF^a^	TGTAGCCGCTAAGCACTACCATCC	15–38	275	This pair of primers are used to clone sequence containing 3a sequence
3a-CR	GGCAACAGCTCAACACCTTTGG	268–289		
3a-F^b^	GGACGGCATCACGATTCTCT	192–211	67	This set of primers and probe are used in real-time PCR reaction of 3a.
3a-R	CCTGAAAACTTGGCAGCAGTT	238–258		
3a-T^c^	Fam-AAACGCCCTCGAATCGCTGGC-Tamra	216–236		
caf1-CF	TTCCGTTATCGCCATTGCAT	15–34	451	This pair of primers are used to clone sequence containing *caf1* sequence
caf1-CR	TGCAAGTTTACCGCCTTTGG	446–465		
caf1-F	AGGTAAACGGTGAGAACCTTGTG	365–387	78	This set of primers and probe are used in real-time PCR reaction of *caf1*.
caf1-R	CAATTGAGCGAACAAAGAAATCC	420–442		
caf1-T	Fam-ATGACGTCGTCTTGGCTACGGGCA-Tamra	392–415		

a CF/CR:short for clone forward primer and clone reverse primer.

b F/R:forward primer and reverse primer.

c T:TaqMan probe.

d FAM: 6-carboxy fluorescein; TAMRA: 6-carboxy-tetra-methylrhodamine.

e Refererring to the positions in the corresponding sequences.

### Real-time PCR and cycling parameters

The PCR system (20µl) contained 2µl of 10×buffer (500 mM KCl, 100 mM Tris-HCl, 25 mM MgCl_2_, 1mg/ml glutin), 1µl of each forward (F) and reverse (R) primer (5µM), 1µl of TaqMan probe (5µM), 1.6 µl of dNTPs (2. 5 mM), 0.2µl of *Taq* DNA polymerase (5U/µl), 5µl of DNA template, 5µl of enzyme stabilizer mixture (40% trehalose and 20% dextran), and 3.2µl of ddH_2_O. Real-time PCRs were all performed on the Roche LightCycler 1.0 with the optimized cycling parameters of pre-denaturation at 94°C for 5 min, 40 cycles of denaturation at 95°C for 5 s, annealing and extension at 60°C for 30 s, and finally cooled at 40°C for 10 s. Signal acquisition mode is “single” at each cycle end of amplification.

### Standard curve

DNA fragments flanking amplicons of 3a and *caf1* were amplified using primers CF and CR (Table 2), respectively, from the 91001 genomic DNA for cloning into the pGEM-T Easy Vector according to the standard protocols reported elsewhere [18],[27]. The ligation products were transformed into DH5α and positive clones were identified by PCR and sequencing. Plasmids containing target fragments were purified separately and linearized by *Sal* I digestion. The concentration of linearized plasmid solution was then determined by UV spectrophotometer for calculating the copy numbers of the target DNAs. The quantified plasmid solution was serially diluted by 10-fold to prepare the standard templates with known copy numbers of target DNAs. Therefore, the real-time PCR was performed using these templates for obtaining the standard curves of C_t_-Log concentration for 3a and *caf1*, respectively [18],[27]. C_t_, the cycle threshold, refers to the cycle at which the fluorescence from a sample crosses the threshold.

### Sensitivity and specificity

*Y. pestis* live attenuated strain EV76 was cultivated in Luria-Bertani broth overnight at 26°C and then serially diluted by 10-fold. The number of viable cells was determined by counting colony forming unit (CFU) on the HBI (Heart Brain Infusion) agar plate. Five µl of each sample were directly applied to PCR amplification for evaluating the sensitivity of the PCR systems. All experiments were performed in duplicate. Genomic DNAs listed in Table 1, human and mouse DNAs were employed to evaluate the specificity of the PCR systems.

### Stability of real-time PCR reagent

All reagents needed for real-time PCR were mixed up at appropriate proportion and dispensed into 200 µl microcentrifuge tubes with 15 µl each for a single reaction. The dispensed reaction mixture was then vacuum-dried. For stability evaluation, the dry reagents were kept away from lights at 37°C up to 79 days and the reagents were taken out at different time points to perform PCR by the following method. The reagents were reconstituted by adding 10 µl of ddH_2_O, and then, 5 µl of plasmid templates containing 10^1^, 10^3^, 10^5^ and 10^7^ copies/µl were separately added. The real-time PCRs were performed according to the protocol mentioned above. Only the real-time PCR system for 3a primers and probe was evaluated in this experiment. The stability of the real-time PCR reagent is assessed in terms of accuracy and reproducibility.

A standard curve of the PCR system was made using the plasmid templates as mentioned above, and the “log_10_ concentration” of the freshly prepared reagents was chosen as reference values. Results of “log_10_ concentration” of the reagents kept at 37°C for different periods were compared with the reference ones for accuracy evaluation.

Four plasmid templates (10^1^, 10^3^, 10^5^, and 10^7^copies/µl) were used to perform PCR in triplicate in parallel. The coefficient of variation (CV) of C_t_ values was used to evaluate the reproducibility of the 3a PCR systems.

### Detection of *Y. pestis* in spiked soil samples

A culture of *Y. pestis* EV76 was serially diluted by 10-fold, and the number of viable cells was determined. For each of serial dilutions, 100 µl was added to 0.2 g soil samples, and the spiked samples were inoculated at 4°C over night for complete adsorption of the bacteria to soil. The soil samples were collected from the yard of our institute, and they were air dried at room temperature. The dried soil samples were directly spiked with dilution of *Y. pestis* cultures without any pretreatment for mimicking the real situation that is usually confronted in practice.

The spiked soil samples were vortexed thoroughly and centrifuged at 2, 000 rpm for 4 min and the supernatants were collected. Then, 1 ml of dH_2_O was added to the pellet, and the mixture was vortexed completely and centrifuged again at 2, 000 rpm for 4 min. The supernatant was transferred to the tube containing the supernatants collected in the first step. This washing step was repeated once for collecting as many bacteria in the soil as possible. The supernatants collected above were mixed together for centrifuging at 8,000 rpm for 5 min to collect the target bacteria. The pellets were washed by dH_2_O once, and 100 µl of dH_2_O was added to each sample to resuspend the pellet for boiling in a water bath for 5 min. Five microlitres of this solution were used as templates for real-time PCR. We evaluated the sensitivity and specificity of the PCR system for detecting *Y. pestis* in the spiked soil samples and a blind experiment was performed for evaluating the feasibility of applying this technique in detection of *Y. pestis* directly from soil samples.

### Sensitivity and specificity for detecting *Y. pestis* in soil samples

The spiked soil samples with different concentrations of *Y. pestis* were prepared and processed following the procedures described above, and the washed supernatants were used for sensitivity evaluation. As for the specificity test, genomic DNA (100 µg each) of the four biovars of *Y. pestis*, and other bacterial genomic DNAs listed in Table 1 (100 µg each) were all spiked into soil samples separately, and the real-time PCR was performed to test the specificity of the primers and probes. All experiments were performed in triplicates.

### Blind detection of *Y. pestis* in the spiked soil samples

Soil samples spiked with different concentrations of EV76 were prepared by our colleague 1, and disordered and renumbered by our colleague 2, and then, the real-time PCR were performed by the first two authors of this article. After reporting the results by the first two authors, the three sides involved in this blind experiment sat together for evaluating agreement of the results to the original preparations. The blind test was performed in triplicates.

## Results

### Standard curves

**Figure 1:**
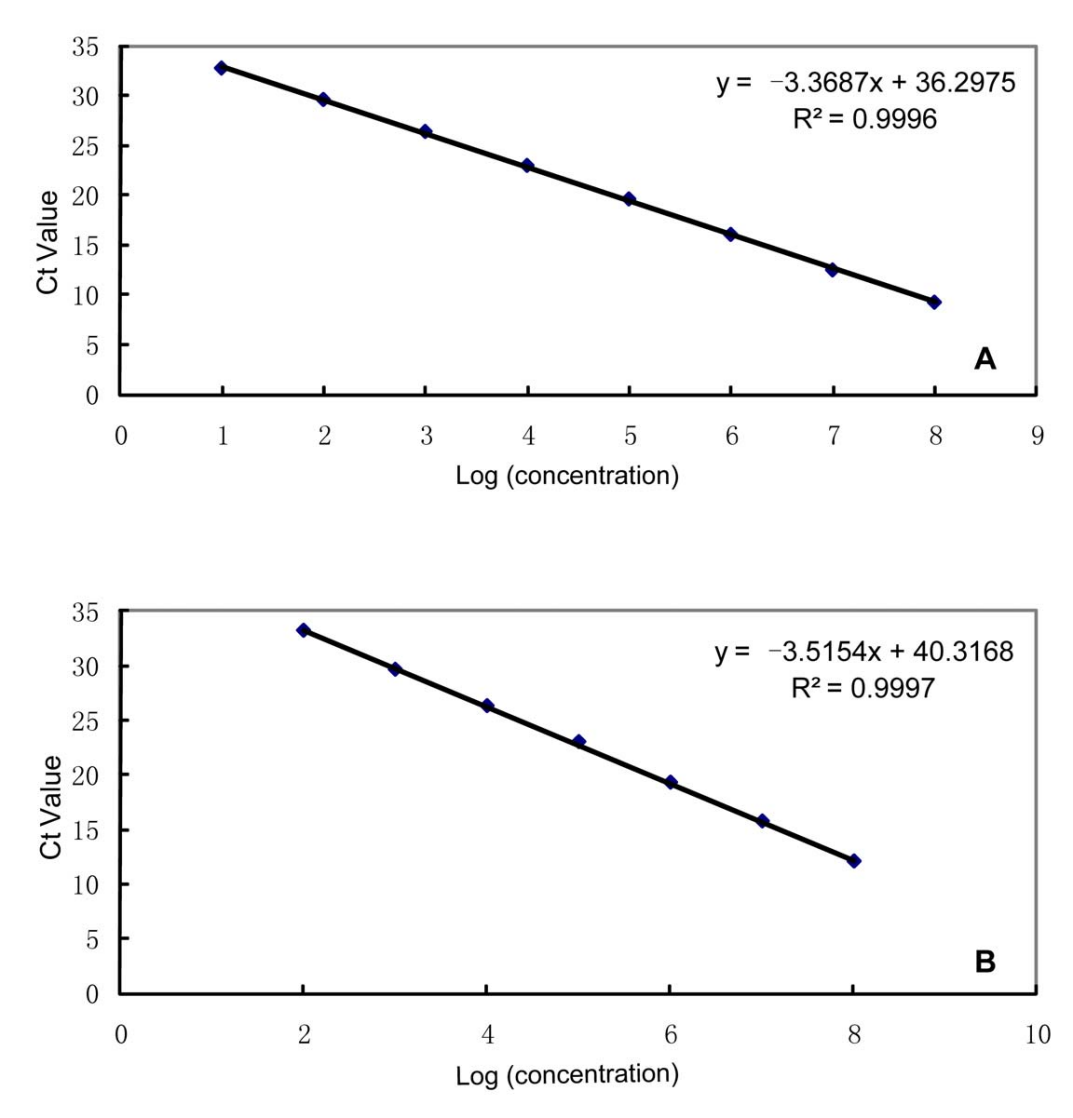
Standard curves for the two targets. Standard curve of using linearized plasmid containing 3a sequence (A) and *caf1* sequence (B) as template. The X-axis represents the log_10_ concentrations of serially diluted template (10–10^8^ copies/µl) and the Y-axis stands for the C_t_ values. Please see the text for detail.

As shown in Fig. 1A and Fig. 1B, standard curves of both 3a and *caf1* sequences, based on 10-fold serial dilutions of 10–10^8^ copies/µl, have a linear relationship between the log_10_ concentration and the C_t_s, with slopes of −3.3687 and −3.5154, respectively. The efficiency of each reaction is 98% and 93%, and Y-intercepts of 36.298 and 40.317, respectively, indicating a good quantitative determination of the PCR system. The r^2^ (coefficient of determination) are 0.9996 and 0. 9997, for 3a- and *caf1*-based PCRs, respectively, implying the accuracy of serial dilution and the precision of sampling, and the ideal linearity of our PCR system as well.

### Sensitivity and specificity

The sensitivity of real-time PCR system based on the 3a and the *caf1* sequences is 1.5 and 15 CFU per system, respectively, using dilutions of EV76 as template (Table 3). For both 3a and *caf1* primers and probes, all four biovars of *Y. pestis* gave positive signals, and the other genomic DNAs in Table 1, human and mouse DNAs gave negative results, indicating the good specificity of these PCR systems.

**Table 3:** Sensitivity test for 3a and *caf1* using live bacteria as template.

	C_t_	Live bacteria concentration (CFU/µl)
Sensitivity test for 3a	34.91	3.03×10^−1^
	31.89	2.81
	27.87	3.14×10
	24.96	3.22×10^2^
	21.75	2.95×10^3^
	18.60	2.85×10^4^
Sensitivity test for *caf1*	32.23	3.23
	29.36	3.25×10
	25.79	2.86×10^2^
	22.98	2.92×10^3^
	19.27	3.16×10^4^
	16.83	3.31×10^5^

a C_t_: threshold cycle; values <35 are considered to be positive.

### Stability of real-time PCR reagent

The vacuum-dried reagents were put in a 37°C incubator and taken out at different time points. We used the ratio of “log_10_ concentration” of them against the original “log_10_ concentration” of the standards as a criterion to evaluate the stability of the reagents. A value close to 1 indicates good stability of the reagents, and we set the ratio of 0.900∼1.100 as the standard of acceptable stability. As shown in Table 4, only the results at the days 18 and 79 for lower concentration (10 copies/µl) were beyond this threshold. However, for the days 23, 32 and 49 at this concentration, the results were good enough to indicate the stability of the reagents. Therefore, we could reasonably attribute the biased results at day 18 for the lower concentration to the experimental errors. For concentration of template higher than 10 copies/µl, the vacuum-dried real-time PCR reagents could remain effective for at least 79 days at 37°C.

**Table 4:** Ratio of every time test results vs. the first test result.

	Log_10_CET/ Log_10_CFT*												
concentration	Day1	Day3	Day5	Day7	Day9	Day11	Day13	Day15	Day18	Day23	Day32	Day49	Day79
10	1	1.03	1.02	1.09	0.94	1.05	0.99	0.97	1.21	1.08	0.95	1.05	1.57
10^3^	1	0.96	0.99	0.97	1.03	0.96	0.93	0.94	0.91	1.00	0.96	0.94	0.90
10^5^	1	0.99	0.99	1.00	1.01	0.96	0.98	1.00	0.97	0.99	0.97	0.98	0.95
10^7^	1	0.98	0.97	0.99	0.98	0.97	0.98	0.96	0.94	0.95	0.96	0.92	0.92

*As the experiments proceeded, a calculated concentration was obtained at each time point for each of the four theoretical concentrations, then, the ratio of Log_10_(concentration measured at each time, CET)/ Log_10_(concentration measured at the first time, CFT) are calculated, each concentration was measured three times. The unit of concentration is copies/µl.

For evaluating the reproducibility of the PCR reagents, four concentrations of standard templates (10, 10^3^, 10^5^, 10^7^copies/µl) were chosen for performing real-time PCR in triplicates. As illustrated in Table 5, the coefficients of variations (CV) of C_t_ value for all concentrations were less than 3% in the 79-day's storage at 37°C, indicating an excellent reproducibility.

**Table 5:** C_t_ value for PCR reagent stored at 37°C for different days.

	C_t_ value^a^													CV^b^
concentration	Day1	Day3	Day5	Day7	Day9	Day11	Day13	Day15	Day18	Day23	Day32	Day49	Day79	
10	31.65	31.5	31.56	31.27	31.92	31.41	31.71	31.76	30.75	31.3	31.86	31.43	29.14	2.40
10^3^	28.1	28.45	28.19	28.3	27.84	28.44	28.67	28.54	28.79	28.11	28.42	28.6	28.9	1.05
10^5^	22.77	22.93	22.94	22.73	22.68	23.27	23.02	22.83	23.13	22.95	23.16	23.08	23.44	0.95
10^7^	15.07	15.49	15.78	15.37	15.55	15.74	15.4	15.82	16.41	16.13	15.97	16.74	16.66	2.97

a C_t_: threshold cycle;values<35 are considered to be positive. Each C_t_ value is the mean result of the three values obtained each time point.

b CV: coefficients of variations.

The results above suggest that the vacuum-dried real-time PCR reagent could be transported at room temperature by normal post or express mailing system.

### Detection of *Y. pestis* in artificially contaminated soil samples

For the spiked soil with different concentrations of *Y. pestis* EV76 for evaluating sensitivity of our PCR system, the results demonstrated that 5×10^4^ CFU per gram of soil could be detected by both 3a- and *caf1*-based PCR systems when C_t_>35 was considered as a negative result. Genomic DNAs of four biovars of *Y. pestis* and its closely related and non-related bacterial genomic DNAs were all spiked separately into soil samples for evaluating the specificity of the PCR systems, and only the samples spiked with the four biovars of *Y. pestis* DNA showed positive results while all the samples spiked with other DNAs gave negative results. In the blind test, all the spiked samples with different amount of *Y. pestis* EV76 were detected correctly. As expected, the CFU values determined by the real-time PCR were 10 to 100 times lower than the ones spiked into the soil samples. These results implied that the real-time PCR systems could detect *Y. pestis* sensitively and specifically from soil samples.

## Discussion

*In vitro* amplification of nucleic acids by PCR has been widely used for both research and clinical diagnosis. Although quantitative real-time PCR of the TaqMan technology has been developed and applied in detection of pathogenic microorganisms from different clinical samples for many years, the reagents or diagnostic kits should be transported under dry ice for protecting enzymes from activity loss. The reverse-transcriptase PCR kit for detecting dengue virus was once evaluated by storing at room temperature, refrigerator and freezer, and the results indicated that the test kits could only be stored above its recommended storage temperature of −20°C for no more than 3 days [28]. Ramanujam et al. reported that the PCR reaction mixtures could be stabilized in carbohydrate polymers by forming glassy matrices that provide room-temperature stability [29]. This kind of stabilized reagent could be stable at 22°C for 6 weeks. Wolff et al. modified a nested PCR system using single-tube, preformulated mixes embedded in a trehalose matrix, which allowed the reagents to be stored for >6 months at ambient temperature [30]. Carbohydrates (trehalose, inulin or dextran) were also employed to stabilize influenza subunit vaccine in the dry state, making the vaccine stable for at least 26 weeks at room temperature [31]. It has been recognized that some yeast cells tend to accumulate high concentrations of trehalose when submitted to heat shock, which can lead to a more heat-stable conformation of the enzyme in yeast [32]. Trehalose has been used to stabilize methanol dehydrogenase for the detection of methanol [33]. In this study, we initially wanted to compare different kinds of carbohydrates in PCR mixture for their ability in stabilizing the PCR reagents. Fortunately, when we tested our first mixture of carbohydrates (10% trehalose and 5% dextran in PCR mixture, final concentration), we obtained very stable PCR reagents as mentioned in the result. Then, we gave up more comparisons between different kinds of carbohydrates and turned to evaluate how stability of this fortunate formulation. During the development of this ambient-stable PCR reagent, we once compared the dried reagents with or without stabilizers. The results showed that the dried reagents without stabilizers could give positive amplifications for higher concentrations of DNA template for the first two days at 37°C (data not shown), however, the one with stabilizers, as shown in this report, could be stable for a much longer period at 37°C. This result indicated the stabilizing effect of the added carbohydrates on PCR reagents.

As shown by our results, this set of reagents can be stable at 37°C for up to 79 days when the concentration of template is higher than 10 copies/µl. For lower concentration, it can be stable at least for 49 days at 37°C. These results reveal that the stabilized PCR reagent can be transported by normal post mailing or express mailing system without dry-ice protection. To test its feasibility of delivering the reagents to different places without cold preservation, we mailed this reagent to different labs in Xinjiang, Shanghai, Tibet, Sichuan, Liaoning, Guangdong, Hubei Provinces by domestic couriers. It usually took 2 to 4 days for the receivers to get the reagent. The receivers tested them and confirmed that the sensitivity of the reagent met the requirement (data not shown).

Plague is one of the most important natural focus-related zoonotic diseases. Routine surveillances are of paramount significance for disease case reporting and controlling. In some of the surveillance stations, dead animals without any information of its dead time and reason and their spoiled soil in the natural plague foci are common samples for detecting and isolating *Y. pestis*. It usually takes 10 to 20 days to get final results. If PCR could be used in routine surveillances, it would improve the surveillance efficiency [12],[34]. However, the surveillance stations are usually set up in the field, some of which even do not have freezers or refrigerators. A room-temperature-stable PCR reagent is therefore urgently demanded. After developing such a kind of reagent and testing its stability at 37°C, we chose the spiked soils as clinical samples to develop a protocol for detecting *Y. pestis* because contaminated soils by dead animals are common samples for *Y. pestis* detection [35] and *Y. pestis* could be persisted in soils for a long time, up to 40 weeks [36]; and also because soils contain rich compounds that inhibit enzymes, including *Taq* polymerase used in PCR [37],[38],[39],[40]. A protocol for soil processing can be readily modified to treat the animal specimens by adding a step of proteinase K digestion. During soil treatment, we once compared the different lysis methods of direct heating lysis and NaI lysis. Direct heating method gave a better sensitivity than NaI lysis and hence we employed the former in our final protocol.

In the future application, we will distribute these reagents to different plague surveillance stations in China for their evaluation of its feasibility in field.
